# Tubal ligation, hysterectomy and ovarian cancer: A meta-analysis

**DOI:** 10.1186/1757-2215-5-13

**Published:** 2012-05-15

**Authors:** Megan S Rice, Megan A Murphy, Shelley S Tworoger

**Affiliations:** 1 Channing Laboratory, Department of Medicine, Brigham and Women’s Hospital and Harvard Medical School, Boston, MA, USA; 2 Department of Epidemiology, Harvard School of Public Health, Boston, MA, USA; 3 Channing Laboratory, 181 Longwood Ave., 3rd Floor, Boston, MA 02115, USA

**Keywords:** Ovarian neoplasms, Sterilization, Tubal, Hysterectomy

## Abstract

**Purpose:**

The purpose of this meta-analysis was to determine the strength of the association between gynecologic surgeries, tubal ligation and hysterectomy, and ovarian cancer.

**Methods:**

We searched the PubMed, Web of Science, and Embase databases for all English-language articles dated between 1969 through March 2011 using the keywords “ovarian cancer” and “tubal ligation” or “tubal sterilization” or “hysterectomy.” We identified 30 studies on tubal ligation and 24 studies on hysterectomy that provided relative risks for ovarian cancer and a p-value or 95% confidence interval (CI) to include in the meta-analysis. Summary RRs and 95% CIs were calculated using a random-effects model.

**Results:**

The summary RR for women with vs. without tubal ligation was 0.70 (95%CI: 0.64, 0.75). Similarly, the summary RR for women with vs. without hysterectomy was 0.74 (95%CI: 0.65, 0.84). Simple hysterectomy and hysterectomy with unilateral oophorectomy were associated with a similar decrease in risk (summery RR = 0.62, 95%CI: 0.49-0.79 and 0.60, 95%CI: 0.47-0.78, respectively). In secondary analyses, the association between tubal ligation and ovarian cancer risk was stronger for endometrioid tumors (summary RR = 0.45, 95%CI: 0.33, 0.61) compared to serous tumors.

**Conclusion:**

Observational epidemiologic evidence strongly supports that tubal ligation and hysterectomy are associated with a decrease in the risk of ovarian cancer, by approximately 26-30%. Additional research is needed to determine whether the association between tubal ligation and hysterectomy on ovarian cancer risk differs by individual, surgical, and tumor characteristics.

## Introduction

Ovarian cancer is the fifth leading cause of cancer death in US women [1], yet primary prevention recommendations are limited. Gynecological surgeries including tubal ligation and hysterectomy may alter ovarian cancer risk by protecting the ovary from ascending carcinogens or damaging the utero-ovarian artery altering hormonal function. In addition, tubal ligation may increase immunity against the surface glycoprotein human mucin 1 (MUC1) [2-4]. While tubal ligation and hysterectomy generally have been found to be inversely associated with ovarian cancer, effect estimates vary between studies and little is known about potential effect modifiers of these associations. Therefore, we conducted a meta-analysis of the association between ovarian cancer and tubal ligation as well as hysterectomy.

## Materials and methods

Through searches in the PubMed, Web of Science, and Embase databases, we sought to identify all English-language articles with quantitative data on the association between tubal ligation or hysterectomy and the risk of ovarian cancer. Database searches encompassed articles dated 1969 through March 2011. We identified articles using the keywords “ovarian cancer” and “tubal ligation” or “tubal sterilization” as well as “ovarian cancer” and “hysterectomy.” In addition, we reviewed the references of selected articles to identify studies missed through our search. We also completed a reverse citation query to include pertinent articles, which referenced those already identified, using the Cited Reference Search application available through the Web of Science. All articles selected for inclusion in our analyses were verified by a second reviewer.

We abstracted relative risks (RRs) and 95% CIs or p-values from selected articles. We used estimates adjusted for multiple confounders when available and calculated standard errors from the 95% CIs or p-values. We decided apriori to use a random-effects model to calculate the summary RR estimates and 95% CIs [5]. Q tests for heterogeneity were used to evaluate the consistency of findings among studies and Begg’s and Egger’s tests were used to assess publication bias [6,7]. We conducted meta-regression analyses to assess whether effect estimates differed by study design (i.e., case–control versus cohort versus other design) and by population studied (i.e., general population versus BRCA mutation carriers) [8]. In secondary analyses, we conducted meta-regression analyses in subsets of the studies to assess whether the effect estimates differed by age at procedure, years since procedure, and, for the tubal ligation analysis, by histological subtype (i.e., serous, mucinous, endometrioid, clear cell, other). All analyses were conducted using the Stata/SE 10.0 for Windows.

## Results

### Database search

**Figure 1:**
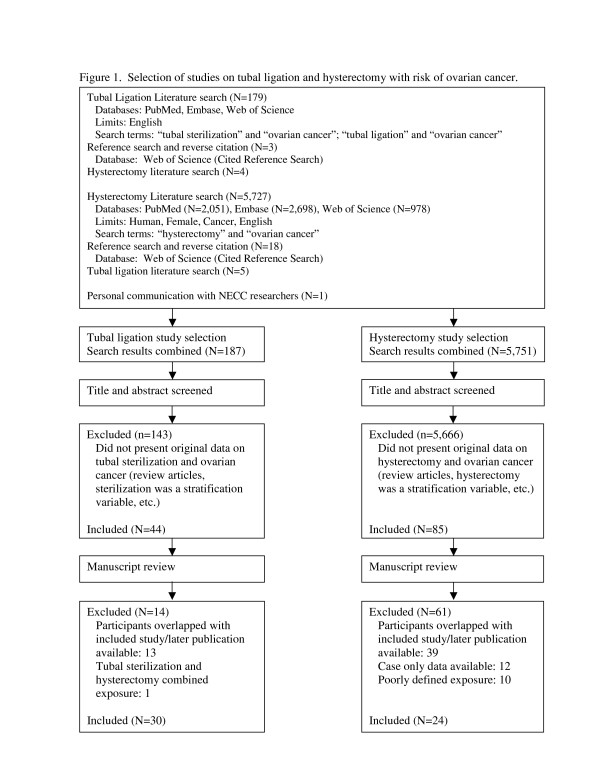
Selection of studies on tubal ligation and hysterectomy with risk of ovarian cancer.

We identified 30 studies that provided estimates of the risk of ovarian cancer in relation to tubal ligation as well as the p-value or 95% confidence interval (CI) [9-37] to include in the meta-analysis (Figure 1). One of the studies examined the risk of ovarian cancer death [28] and three studies were conducted in BRCA carriers [13,18,20]. Therefore, we conducted sensitivity analyses examining the influence of these studies, which are detailed below. For the examination of hysterectomy and ovarian cancer, we identified 24 studies to include in the meta-analysis (Figure 1) [9,10,12,13,15,16,23-26,29,31,32,38-47]. Nine of the studies reported effect estimates for simple hysterectomy, [23,25,29,32,38,42,43,45] seven provided estimates for hysterectomy with unilateral oophorectomy, [23,29,32,38,42,45] and 15 did not distinguish whether or not women with hysterectomy underwent a unilateral oophorectomy [9,10,12,13,15,16,24,26,31,39-41,44,46,47]. Two of the studies included in the primary meta-analysis for both tubal ligation and hysterectomy were pooled analyses [9,31], one was comprised of eight studies [31] and another was comprised of four studies [9]. For these studies, we included the pooled estimates in our meta-analysis as we were unable to obtain the study-specific effect estimates for all studies through our literature search. One of the studies identified in our tubal ligation and hysterectomy literature searches was a study in the New England case–control study (NECC) [Cramer]. However, in this study the reference category for the odds ratios for tubal ligation and hysterectomy was comprised of women who did not have any pelvic surgeries, including cesarean sections. In order for the effect estimates from the NECC to be comparable to other studies, we requested and obtained from NECC researchers the odds ratio for ovarian cancer comparing women who had a tubal ligation to those who did not have the procedure as well as the odds ratio comparing women with hysterectomy to those who did not have a hysterectomy. We also obtained odds ratios for the secondary analyses described below.

In secondary analyses, we identified studies that reported the relative risk of ovarian cancer by characteristics of surgery, such as age at or years since procedure, as well as by histological subtype of ovarian cancer. We identified eight studies that reported stratum-specific estimates of ovarian cancer risk by years since tubal ligation (Additional file 1: Table S1) [14,19,25,26,28,29,48] and nine studies that reported stratum-specific estimates for age at tubal ligation (Additional file 1: Table S2) [13,14,19,25,27-29,48]. In addition, 13 studies specified effect estimates for invasive ovarian cancer [10,12,15,17-23,31,33] and 11 studies on tubal ligation reported estimates for at least one histological subtype of ovarian cancer (Additional file 1: Table S3) [9,10,15,16,19,22,24,26,29,49]. Eight studies on hysterectomy reported stratum-specific estimates of ovarian cancer risk by years since the procedure (Additional file 1: Table S4) [25,26,29,31,43,45,46] and five studies reported stratum-specific estimates for age at hysterectomy (Additional file 1: Table S5) [25,29,31,43]. In addition, nine studies reported effect estimates for invasive ovarian cancer [[10,12,15,23,31,40-42], Cramer].

Separate analyses were performed examining risk of ovarian cancer and characteristics of surgery, including years since and age at procedure. For six of the eight studies reporting stratum-specific estimates for years since tubal ligation, we were able to derive estimates for less than 10 years since tubal ligation and 10 or more years since tubal ligation [19,25,26,29,48]. For seven of the nine studies that reported risks by age at tubal ligation, we were able to derive estimates for age less than 35 at tubal ligation and 35 years of age or older [13,19,27-29,48]. For seven of the eight studies reporting stratum-specific estimates for years since hysterectomy, we were able to derive estimates for less than 10 years since hysterectomy and 10 or more years since hysterectomy [22,25,26,31,43,45]. For the five studies that reported risks by age at hysterectomy, we were able to derive estimates for age less than 40 or 45 at hysterectomy and 40 or 45 years of age or older [25,29,31,43] [NECC].

### Tubal ligation

**Figure 2:**
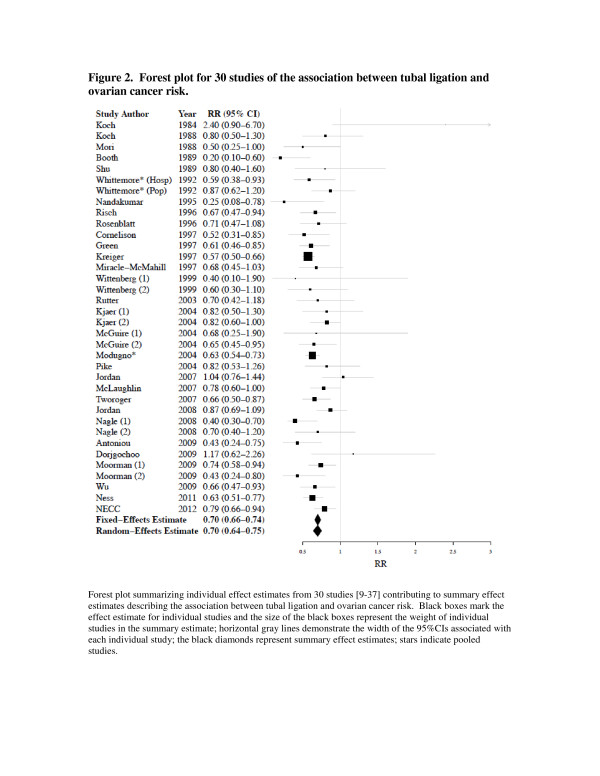
**Forest plot for 30 studies of the association between tubal ligation and ovarian cancer risk.** Forest plot summarizing individual effect estimates from 30 studies [9-37] contributing to summary effect estimates describing the association between tubal ligation and ovarian cancer risk. Black boxes mark the effect estimate for individual studies and the size of the black boxes represent the weight of individual studies in the summary estimate; horizontal gray lines demonstrate the width of the 95%CIs associated with each individual study; the black diamonds represent summary effect estimates; stars indicate pooled studies.

The estimated RRs for ovarian cancer associated with tubal ligation versus no tubal ligation ranged from 0.2 to 2.4 (Table 1). Twenty-seven of the 30 studies reported lower risks of ovarian cancer in women who had a tubal ligation compared to those who had not had the procedure. The three studies that observed an elevated risk of ovarian cancer did not achieve statistical significance [14,16,35]. The summary RR was 0.70 (95%CI: 0.64, 0.75), demonstrating a statistically significant inverse association between tubal ligation and ovarian cancer (Figure 2). Some studies in our analysis did not specify whether borderline cases were included in the analyses. However, when we restricted our analysis to 13 studies that reported the association for invasive ovarian cancer, specifically the summary RR was very similar (summary RR = 0.72; 95%CI: 0.66, 0.72). Since there was evidence of heterogeneity among the 30 studies (*P* = 0.02), we examined the contribution of study characteristics to the heterogeneity. We did not observe statistically significant evidence of heterogeneity by study design (i.e., cohort study, case–control study, or other) or residence of study participants (i.e., USA or non-USA) (P > 0.05) (Table 2). Interestingly, the relative risk among BRCA carriers (RR = 0.64, 95%CI: 0.43-0.96) was similar to the relative risk among population-based studies (RR = 0.70, 95%CI: 0.64-0.76) (Table 2). Overall, we found that if any single study was removed from the meta-analysis, the effect estimate did not change substantially (data not shown). In addition, we found no evidence of publication bias using either the Begg (*P* = 0.12) or the Egger (*P* = 0.22) method for assessing bias.

**Table 1:** Epidemiologic Studies of the Association Between Tubal Ligation and Risk of Ovarian Cancer

**Author (Country)**	**Study Design**	**Case definition**	**Covariates**	**OR, RR, or SIR (95%CI)**	**Comments**
NECC 2012 (USA) [personal communication with Dr. Daniel Cramer]	Case-control	Borderline or invasive epithelial ovarian cancer N=2076	age, study center, BMI , study phase, smoking, family history of ovarian and breast cancers, talc use, OC use, parity, breast feeding, age at menarche, post-menopausal status, use of post-menopausal hormones, hysterectomy	0.79 (0.66-0.94)	
Ness et al. 2011 (USA) [11]	Case-control	Invasive or borderline epithelial ovarian cancer	Age, number of pregnancies, race, infertility, family history of	0.63 (0.51-0.77)	
		N=867	ovarian cancer, ever use of oral contraceptives, ever use of IUDs, ever use of barriers, vasectomy		
Moorman et al. 2009 (USA) [12]	Case-control North Carolina Ovarian Cancer Study	Invasive epithelial ovarian cancer	Age, parity, age at menarche, duration of OC use, family history of breast/ovarian cancer, BMI	Whites: 0.74 (0.58, 0.94)	
				African-Americans: 0.43 (0.24, 0.80)	
		N=746 White cases			
		N=111 African-American cases			
Antoniou et al. 2009 (Europe and Canada) [13]	Retrospective Cohort	Ovarian cancer (only BRCA 1/2 carriers)	Age, duration of OC use, parity	BRCA 1/2: 0.43 (0.24, 0.75)	Includes prevalent and incident cases.
				BRCA1: 0.42 (0.22, 0.80)	
		N=201 BRCA1 cases			
				BRCA2: 0.47 (0.18, 1.21)	Mean difference between age at diagnosis and interview: 6.7 years
		N=52 BRCA2 cases			

Wu et al. 2009 (USA) [37]	Case-control	Invasive and borderline ovarian cancer	Race/ethnicity, age, education, family history of ovarian cancer, menopausal status, use of oral contraceptives, parity	0.66 (0.47, 0.93)	
		N=609 cases			
Dorjgochoo T. et al. 2009 (China) [14]	Prospective cohort	Ovarian cancer	Age, education, age at menarche, parity, breastfeeding, BMI, physical activity, smoking, menopausal status, family history of cancer, other contraceptive methods.	1.17 (0.62, 2.26)	Cohort N=66,661
		N=94 cases			
					76.1% participation rate
Nagle et al. 2008 (Australia) [15]	Case-control	Invasive epithelial endometrioid and clear cell ovarian cancer	Age, education, parity, and hormone contraceptive use	Endometrioid: 0.4 (0.3, 0.7)	47% participation rate in controls
				Clear cell: 0.7 (0.4, 1.2)	
		N=142 endometrioid cases			
		N=90 clear cell cases			
Jordan et al. 2008 (Australia) [10]	Case-control	Invasive epithelial serous ovarian cancer	Parity, hormonal contraceptive use, history of breast or ovarian cancer, age, education	Serous (invasive): 0.87 (0.69-1.09)	
		N=627 cases			
Jordan et al. 2007 (Australia) [16]	Case-control	Epithelial benign serous tumors (N=230) and benign mucinous tumors (N=133)	Age, state of residence, education, parity, hormonal contraceptive use, hysterectomy, smoking status	Combined: 1.04 (0.76-1.44)	65% participation rate in cases, 47% in controls.
				Mucinous: 1.00 (0.61-1.64)	
				Serous: 1.08 (0.75-1.57)	
Tworoger et al. 2007 (USA) [17]	Prospective cohort	Incident invasive epithelial ovarian cancer	Age, BMI, parity, smoking history, age at menarche, age at menopause, duration of postmenopausal hormone use, duration of oral contraceptive use	0.66 (0.50, 0.87)	Update of Hankinson et al. 1993
		N=612 cases			
http://McLaughlin JR et al. 2007 (International) [18]	Case-control	Invasive ovarian cancer (only BRCA 1/2 carriers)	Age, mutation type, country of residence, parity, breastfeeding, oral contraceptive use, ethnicity.	BRCA1+2 carriers: 0.78 (0.60, 1.00)	Includes prevalent and incident cases. Results similar when restricted to women interviewed within 3 years of diagnosis.
				BRCA1: 0.80 (0.59, 1.08)	
		N=799 cases		BRCA2: 0.63 (0.34, 1.15)	
		BRCA1 N=670 BRCA2 N=128			
		BRCA1/2 N=1			
Modugno et al. 2004 (USA) [9]	Pooled case-control	Epithelial ovarian cancer	Study site, age, family history, duration of oral contraceptive use, parity	0.63 (0.54, 0.73)	Pooled analysis from four studies.
		N=2098 cases			
Kjaer et al. 2004 (Denmark) [19]	Population-based follow-up study	Invasive ovarian cancer and borderline ovarian tumor	Age and calendar year	Invasive: 0.82 (0.6, 1.0)	Observed number of cancer cases in cohort of women who underwent tubal ligation was compared to the expected number of cases based on the age and calendar year specific rates from the Danish Cancer Registry.
				Borderline: 0.82 (0.5, 1.3)	
		N=75 invasive cases			
		N=21 borderline cases			
McGuire et al. 2004 (USA) [20]	Case-control	Invasive epithelial ovarian cancer	Age, parity, duration of OC use, race/ethnicity	BRCA 1 carriers: 0.68 (0.25, 1.90)	
				Noncarriers: 0.65 (0.45, 0.95)	
		N=36 BRCA1 cases			
		N=381 noncarrier cases			
Pike et al. 2004 (Los Angeles, USA) [21]	Case-control	Invasive ovarian cancer	Age, ethnicity, SES, education, family history of ovarian cancer, use of talc, BMI, parity, age at last birth, number of incomplete pregnancies, OC use, menopausal status, age at menopause, hormone replacement therapy	0.82 (0.53-1.26)	
		N=477 cases			
Rutter et al. 2003 (Israel) [23]	Case-control	Invasive epithelial ovarian cancer or primary peritoneal cancer	Age, ethnicity, parity, years of oral contraceptive use	0.70 (0.42, 1.18)	Participation rate was 79% for case patients and 66% for controls.
		N=1124 cases			
Wittenberg et al. 1999 (USA) [24]	Case-control	Mucinous and non-mucinous epithelial ovarian cancer	Age at diagnosis, parity, duration of OC use	Mucinous: 0.4 (0.1, 1.9)	64% participation rate in cases, 72% in controls. Included both borderline and invasive.
				Non-mucinous: 0.6 (0.3, 1.1)	
		N=43 mucinous cases			
		N=279 non-mucinous cases			
Kreiger et al, 1997 (Canada) [25]	Historical cohort study	Invasive and borderline ovarian cancer	Age, calendar year, length of follow-up	0.57 p<0.001	Calculated observed over expected events.
		N=108 observed cases in tubal ligation subcohort			Sensitivity analysis excluding borderline malignancies similar.
Green, Purdie, et al. 1997 (Australia) [26]	Case-control	Incident, primary epithelial ovarian cancer	Age, education, BMI, parity, OC duration, smoking, family history of ovarian cancer	0.61 (0.46, 0.85)	90% participation rate in cases, 73% in controls.
		N=824 cases			
Cornelison et al 1997 (USA) [27]	Case-control	Ovarian cancer N=300 cases	Age , SES, marital status, parity, age at first pregnancy, age at menarche, age at menopause, irregular menses, breast-feeding duration, BMI, OC use	0.52 (0.31,0.85)	Patient controls with no malignancy or ovarian disease.
Miracle-McMahill, et al. 1997 (USA) [28]	Prospective Cohort Study	Ovarian cancer mortality	Age, race, BMI, education, family history of ovarian cancer, family history of breast ca, parity, marital status, age at menarche, OC use, ERT, age at menopause, miscarriages smoking status	0.68 (0.45, 1.03)	
		N=799 ovarian cancer deaths			
Rosenblatt, et al. 1996 (International) [29]	Case-control	Borderline or malignant epithelial ovarian cancer	Age, hospital, year of interview, parity OC use	0.71 (0.47, 1.08)	No differences observed for borderline and malignant tumors.
		N=385 cases			
Risch et al. 1996 (Canada) [22]	Case-control	Epithelial ovarian cancer	Age, parity, years of OC use, average lactation/pregnancy, total years of ERT, hysterectomy, family history of breast cancer	0.67 (0.47-0.94)	Invasive and borderline tumors included.
		N=450 cases Borderline			
		N=83 Invasive N=376			
Nandakumar et al. 1995 (India) [30]	Case-control	Ovarian cancer	Age, residential area, parity, age at first birth	0.25 (0.08, 0.78)	Restricted to ever-married women. Hospital-based controls.
		N=97 cases			
Whittemore et al 1992 (USA) [31]	Pooled case-control	Invasive epithelial ovarian cancer	Age, study, parity, OC use	Hospital-based studies:	Restricted to white women. 6 hospital based studies and 6 population-based studies.
				0.59 (0.38, 0.93) Population-based studies: 0.87 (0.62, 1.20)	

		N=2197 cases			
Booth et al 1989 (England) [32]	Case-control	Epithelial ovarian cancer	Age, social class, gravidity, unprotected intercourse	0.2 (0.1, 0.6)	Cases were less than 65 years old and interviewed within 2 years of diagnosis. Age-matched hospital-based controls.
		N=235 cases			
Shu et al 1989 (China) [33]	Case-control	Invasive epithelial ovarian cancer	Age, education, parity, age at menarche, ovarian cyst	0.8 (0.4, 1.6)	89% participation rate in cases, 100% in controls. All <70 years of age.
		N=172 cases			
Koch et al 1988 (Canada) [34]	Case-control	Epithelial ovarian cancer	None	0.8 (0.5, 1.3)	47% participation rate in controls. Age-matched, but did not control for age in analyses.
		N=200 cases			
Mori et al 1988 (Japan) [36]	Case-control	Primary epithelial ovarian cancer	Age, parity, marital status, number of induced abortions	0.5 (0.25, 1.00)	Controls were hospital in-patients with gynecological complaints other than ovarian cancer and OB/GYN outpatients without a malignant ovarian disorder. 100% participation rate in cases and controls.
		N=110 cases			
Koch et al. 1984 (Canada) [35]	Retrospective cohort	Ovarian cancer N=4 cases	Age, nulliparity	2.4 (0.9, 6.7)	Population who underwent tubal ligation were mental patients. 34% were lost to follow-up. Many underwent the procedure at young ages (i.e. 10-19). Expected rates calculated from a previous retrospective study. Incomplete adjustment for parity.

Abbreviations: OR, odds ratio; RR, relative risk; SIR, standardized incidence ratio; OC, oral contraceptive; BMI, body mass index; SES, socio-economic status; ERT, estrogen replacement therapy.

**Table 2:** Summary relative risks for tubal ligation and ovarian cancer by selected characteristics

	Number of contributing studies	Random-effects RR (95%CI)
Study design	30 studies	
Cohort study		0.67 (0.50, 0.90)
Case-control study		0.70 (0.63, 0.75)
Other study design		0.95 (0.63, 1.43)
BRCA status	30 studies	
BRCA positive		0.64 (0.43, 0.96)
General population		0.70 (0.64, 0.76)
Geographic location	30 studies	
US		0.68 (0.63, 0.73)
Non-US		0.71 (0.61, 0.82)
Histologic subtype	11 studies	
Serous		0.75 (0.65,0.88)
Endometrioid		0.45 (0.33,0.61)
Mucinous		0.88 (0.70,1.09)
Clear cell		0.72 (0.55,0.94)
Other		0.80 (0.63,1.01)
Age at tubal ligation	7 studies	
<35 years of age		0.69 (0.59,0.81)
35+ years of age		0.79 (0.68,0.92)
Years since tubal ligation	6 studies	
<10 years		0.69 (0.59,0.79)
10+ years		0.68 (0.54,0.87)

Eight of the studies examined years since tubal ligation. In a meta-regression of six of these studies, we did not observe a difference in the relative risk of ovarian cancer between women who had a tubal ligation less than 10 years ago (summary RR = 0.69, 95%CI: 0.59, 0.79) and those women who had a tubal ligation 10 or more years ago (summary RR = 0.68, 95%CI: 0.54, 0.87) (*P*-heterogeneity = 0.78) (Table 2). Of the other studies, a prospective cohort study of ovarian cancer mortality reported tubal ligation to be associated with a reduced risk for women who had the procedure within 20 years, with a smaller non-significant reduced risk for those who had the procedure 20 or more years ago.[19] However, a prospective cohort study based in China observed a non-significant increase in risk that was similar for both women who had a tubal ligation less than 33 years ago and women who had a tubal ligation 33 or more years ago [14].

Nine studies examined age at tubal ligation on ovarian cancer risk. In a meta-regression of seven of these studies, the relative risk for ovarian cancer was non-significantly lower among women who had a tubal ligation when they were younger than 35 (summary RR = 0.69, 95%CI: 0.59, 0.81) compared to at 35 years of age or older (summary RR = 0.79, 95%CI: 0.68, 0.92), although the difference was not statistically significant (*P-for-heterogeneity* = 0.22) (Table 2). In addition, the Shanghai Women’s Health Study noted a non-significant increase in ovarian cancer risk only among women who were less than 30 when they underwent the procedure and no association among those aged 30 or more at time of surgery [14]. In a historical cohort study, tubal ligation was associated with a reduced risk of ovarian cancer among women aged 25–44 at time of the procedure (RR = 0.54, p < 0.001), but not among women aged 45–64 at the time of their tubal ligation (RR = 1.18, p = 0.68) [25].

Eleven studies reported effect estimates by at least one histologic subtype. In a meta-analysis regression we observed that the association was stronger for endometrioid tumors compared to serous tumors (*P* < 0.01). The summary RR for serous tumors was 0.75 (95%CI: 0.65, 0.88) compared to 0.45 (95%CI: 0.33, 0.61) for endometrioid tumors. The summary RRs for mucinous (summary RR = 0.88, 95%CI: 0.70,1.09), clear cell (summary RR = 0.72, 95%CI: 0.55, 0.94), and other tumor types (summary RR = 0.80, 95%CI: 0.63,1.01) did not significantly differ from serous tumors (p > 0.05).

### Hysterectomy

**Figure 3:**
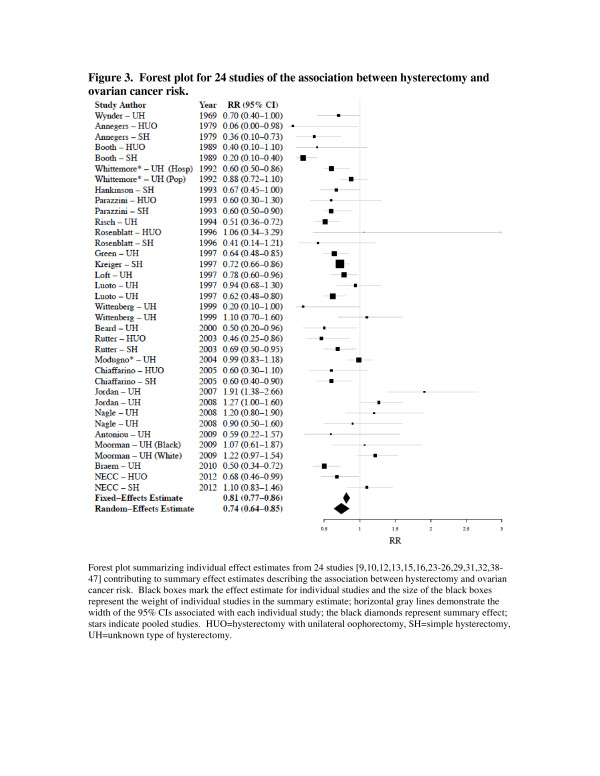
**Forest plot for 24 studies of the association between hysterectomy and ovarian cancer risk.** Forest plot summarizing individual effect estimates from 24 studies [9,10,12,13,15,16,23,26,29,31,32,38-47] contributing to summary effect estimates describing the association between hysterectomy and ovarian cancer risk. Black boxes mark the effect estimate for individual studies and the size of the black boxes represent the weight of individual studies in the summary estimate; horizontal gray lines demonstrate the width of the 95% CIs associated with each individual study; the black diamonds represent summary effect; stars indicate pooled studies. HUO=hysterectomy with unilateral oophorectomy, SH=simple hysterectomy, UH=unknown type of hysterectomy.

The study-specific RRs for ovarian cancer associated with hysterectomy (with or without unilateral oophorectomy) ranged from 0.06 to 1.91 (Table 3). The summary RR was 0.74 (95%CI: 0.65, 0.84), demonstrating a statistically significant inverse association between hysterectomy and ovarian cancer (Figure 3). When we restricted to nine studies that reported effect estimates for invasive ovarian cancer, the association was similar (summary RR = 0.81; 95%CI: 0.68, 0.97). We also calculated summary estimates for simple hysterectomy and hysterectomy with unilateral oophorectomy (Table 4). We observed that the reduced risk of ovarian cancer associated with hysterectomy with unilateral oophorectomy (RR = 0.60, 95%CI: 0.47-0.78) was similar to the reduced risk associated with simple hysterectomy (RR = 0.62, 95%CI: 0.49-0.79). We examined the contribution of other study characteristics to the heterogeneity between studies, since the p-heterogeneity <0.01. We did not observe evidence for statistically significant heterogeneity by study type (i.e., case–control, cohort, other) or geographic location (i.e., USA vs non-USA) (*P* > 0.05) (Table 4). Overall, if any single study was removed from the meta-analysis, the effect estimate did not change substantially (data not shown). We did note evidence of publication bias using the Egger (*P* = 0.01) method for assessing bias, but not for the Begg method (P = 0.11).

**Table 3:** Epidemiologic Studies of the Association Between Hysterectomy and Risk of Ovarian Cancer

**Author (Country)**	**Study Design**	**Case definition**	**Covariates**	**OR, RR, or SIR (95%CI)**	**Comments**
NECC 2012 (USA) [Personal communication with Dr. Daniel Cramer]	Case-control	Borderline and invasive ovarian cancer	age, study center, BMI , study phase, smoking, family history of ovarian and breast cancers, talc use, OC use , parity, breast feeding, age at menarche, post-menopausal status, use of post-menopausal hormones, tubal ligation	Hysterectomy only: 1.10 (0.83-1.46)	NECC 2012 (USA) [Personal communication with Dr. Daniel Cramer]
		N=2076		Hysterectomy with unilateral oophorectomy: 0.68 (0.46-0.99)	
Annegers et al. 1979 (USA) [38]	Case-control (Rochester Project)	Epithelial ovarian cancer N=116 cases	Controls matched on age and residence	Hysterectomy only: 0.36 (0.10-0.73)	
				Hysterectomy with unilateral oophorectomy: 0.06 (0.004-0.98)	
Antoniou et al. 2009 (Europe and Canada) [13]	Retrospective Cohort	Ovarian cancer (only BRCA 1/2 carriers)	Age, duration of OC use, parity	Hysterectomy with or without unilateral oophorectomy: BRCA 1/2: 0.59 (0.22, 1.57)	Includes prevalent and incident cases.
		N=201 BRCA1 cases			Mean difference between age at diagnosis and interview: 6.7 years
		N=52 BRCA2 cases			
				BRCA1:0.68 (0.22, 2.12)	
				BRCA2: 0.35 (0.08, 1.58)	
Beard et al. 2000 (USA) [40]	Case-control (Rochester Project)	Invasive epithelial ovarian cancer	Controls matched on age and provider	Hysterectomy with or without unilateral oophorectomy: 0.5 (0.2–0.96)	
		N=103 cases			
Booth et al 1989 (England) [32]	Case-control	Epithelial ovarian cancer	Age and social class	Hysterectomy only: 0.2 (0.1-0.4)	Cases less than 65 years old and diagnosed within 2 years. Age-matched hospital-based controls.
		N=235 cases			
				Hysterectomy with unilateral oophorectomy: 0.4 (0.1-1.1)	
Braem et al. 2010 (Netherlands) [41]	Case-cohort study (Netherlands Cohort Study)	Invasive epithelial ovarian cancer	Age, OC use, parity	Hysterectomy with or without unilateral oophorectomy: 0.50 (0.34-0.72)	All women presumed to be postmenopausal
		N=375			
Chiaffarino et al. 2005 (Italy) [42]	Multi-center case-control study	Incident invasive epithelial ovarian cancer	Age, center, education, parity, OC use, family history of ovarian and breast cancer	Hysterectomy only: 0.6 (0.4-0.9) Hysterectomy and unilateral oophorectomy: 0.6 (0.3-1.1)	
		N=1031 cases			
Green, Purdie, et al. 1997 (Australia) [26]	Case-control	Incident, primary epithelial ovarian cancer	Age, education, BMI, parity, OC duration, smoking, family history of ovarian cancer	Hysterectomy with or without unilateral oophorectomy: 0.64 (0.48-0.85)	90% participation rate in cases, 73% in controls.
		N=824 cases			
Hankinson et al. 1993 (USA) [43]	Cohort study (NHS)	Borderline and malignant epithelial ovarian cancer	Age, parity, duration of OC use, age at menarche, tubal ligation, smoking status, BMI	Hysterectomy only: 0.67 (0.45-1.00)	90% follow-up rate
		N=260 cases			
Jordan et al. 2008 (Australia) [10]	Case-control	Invasive epithelial serous ovarian cancer	Parity, hormonal contraceptive use, history of breast or ovarian cancer, age, education	Hysterectomy with or without unilateral oophorectomy:	
		N=627 cases			
				Serous (invasive): 1.27 (1.00, 1.60)	
Jordan et al. 2007 (Australia) [16]	Case-control	Benign serous tumors (N=230) and benign mucinous tumors (N=133)	Age, state of residence, education, parity, hormonal contraceptive use, smoking status	Hysterectomy with or without unilateral oophorectomy:	65% participation rate in cases, 47% in controls.
					For serous tumors by surgical indication:
				Combined: 1.91 (1.38-2.66)	
				Mucinous: 0.95 (0.55-1.67)	Non-hormonal: 1.1 (0.5-2.7)
				Serous: 2.75 (1.90-3.96)	Hormonal: 3.0 (2.1-4.5)
Kreiger et al. 1997 (Canada) [25]	Historical cohort study	Ovarian cancer N=169 observed cases in hysterectomy subcohort	Age, calendar year, length of follow-up	Hysterectomy only: 0.72 p<0.001	Calculated observed over expected events.
					Sensitivity analysis excluding borderline malignancies similar.
Loft et al. 1997 (Denmark) [44]	Prospective historical cohort study	Ovarian cancer	Age	Hysterectomy with and without unilateral oophorectomy: 0.78 (0.60-0.96)	N=22,135 women w/ hysterectomy (3940 of whom had unilateral oophorectomy)
		N=71			
					Follow-up=12.5 years
Luoto et al. 1997 (Finland) [39]	Historical cohort study	Ovarian cancer	Adjusted for education, parity, and follow-up. Non-hysterectomized women had similar distributions of age and municipality.	Partial hysterectomy: RR=0.94 (0.68-1.30)	Ovarian status not assessed.
		N=53 cases with partial hysterectomy			
		N=91 cases with total hysterectomy			
				Total hysterectomy: RR=0.62 (0.48-0.80)	
Modugno et al. 2004 (USA) [9]	Pooled case-control	Epithelial ovarian cancer	Study site, age, family history, duration of oral contraceptive use, parity, endometriosis, tubal ligation	Hysterectomy with or without unilateral oophorectomy: 0.99 (0.83-1.18)	Pooled analysis from four studies.
		N=2098 cases			Analyzed by endometriosis status.
Moorman et al. 2009 (USA) [12]	Case-control North Carolina Ovarian Cancer Study	Invasive epithelial ovarian cancer	Age, parity, age at menarche, duration of OC use, family history of breast/ovarian cancer, BMI	Hysterectomy with or without unilateral oophorectomy:	
		N=746 White cases			
		N=111 African-Am cases			
				Whites: 1.22 (0.97, 1.54) African-	
				Americans: 1.07 (0.61, 1.87)	
Nagle et al. 2008 (Australia) [15]	Case-control	Invasive epithelial endometrioid and clear cell ovarian cancer	Age, education, parity, and hormone contraceptive use	Hysterectomy with or without unilateral oophorectomy:	47% participation rate in controls
		N=142 endometrioid cases			
				Endometrioid: 1.2 (0.8, 1.9)	
				Clear cell: 0.9 (0.5, 1.6)	
		N=90 clear cell cases			
Parazzini et al. 1993 (Italy) [45]	Case-control study	Epithelial ovarian cancer	Age, education, parity, oral contraceptive use, menarche, menopause	Hysterectomy only: 0.6 (0.5-0.9)	
		N=953 cases		Hysterectomy with unilateral	
				oophorectomy: 0.6 (0.3-1.3)	
Risch et al. 1994 (Canada) [46]	Case-control	Epithelial ovarian cancer	Age, duration of OC use, number of full-term pregnancies	Hysterectomy with or without unilateral oophorectomy: 0.51 (0.36-0.72)	
		N=450 cases			
Rosenblatt et al. 1996 (Multi-national) [29]	Case-control (Multi-site/country)	Borderline or invasive epithelial ovarian cancer	Age, date of diagnosis, center, parity, OC use	Hysterectomy only: 0.41 (0.14-1.21)	
		N=385 cases		Hysterectomy with unilateral oophorectomy: 1.06 (0.34-3.29)	
				Combined: 0.58 (0.27-1.28)	
Rutter et al. 2003 (Israel) [23]	Case-control	Epithelial ovarian cancer or primary peritoneal cancer	Age, ethnicity, parity, years of oral contraceptive use	Hysterectomy only: 0.69 (0.50-0.95)	Participation rate was 79% for case patients and 66% for controls. Includes BRCA-specific analysis.
		N=1124 cases		Hysterectomy with unilateral oophorectomy: 0.46 (0.25-0.86)	
Whittemore et al 1992 (USA) [31]	Pooled case-control (12 studies included)	Invasive epithelial ovarian cancer	Age, study, parity, OC use	Hysterectomy with or without unilateral oophorectomy: Hospital-based studies: 0.66 (0.50-0.86)	Restricted to white women. 6 hospital based studies and 6 population-based studies. All hysterectomies performed at least 2 years prior to reference date.
		N=2197 cases			
				Population-based studies: 0.88 (0.72-1.1)	
Wittenberg et al. 1999 (USA) [24]	Case-control	Mucinous and non-mucinous epithelial ovarian cancer	Age at diagnosis, parity, duration of OC use	Hysterectomy with or without unilateral oophorectomy: Mucinous: 0.2 (0.1, 1.0)	64% participation rate in cases, 72% in controls. Included both borderline and invasive.
		N=43 mucinous cases			
		N=279 non-mucinous cases		Non-mucinous: 1.1 (0.7, 1.6)	
Wynder et al. 1969 (USA) [47]	Case-control (Hospital based)	Epithelial ovarian cancer (N=150) plus miscellaneous ovarian tumors (N=8)	Age-matched controls	Hysterectomy with or without unilateral oophorectomy: 0.7 (0.04-1.0)	

Abbreviations: OR, odds ratio; RR, relative risk; SIR, standardized incidence ratio; OC, oral contraceptive; BMI, body mass index; SES, socio-economic status.

**Table 4:** Summary relative risks for hysterectomy and ovarian cancer by selected characteristics

	Number of contributing studies	Random-effects RR (95%CI)
Study design	24 studies	
Cohort study		0.73 (0.63, 0.85)
Case-control study		0.73 (0.62, 0.86)
Geographic location	24 studies	
US		0.81 (0.67, 0.97)
Non-US		0.70 (0.59, 0.84)
Type of hysterectomy	24 studies	
With unilateral oophorectomy		0.60 (0.47, 0.78)
Without oophorectomy		0.62 (0.49, 0.79)
Unknown oophorectomy		0.83 (0.71, 0.98)
Age at hysterectomy	5 studies	
<40/45 years of age		0.70 (0.55, 0.89)
40/45+ years of age		0.83 (0.72, 0.96)
Years since hysterectomy	7 studies	
<10 years		0.69 (0.60, 0.79)
10+ years		0.77 (0.66, 0.89)

Eight studies examined years since hysterectomy and ovarian cancer risk. In a meta-regression of seven of these studies, the RR of ovarian cancer between women who had the procedure 10 or more years ago was slightly lower compared to women who had a hysterectomy less than 10 years ago (summary RR = 0.69, 95%CI: 0.60, 0.79 and summary RR = 0.77, 95%CI: 0.66, 0.89 respectively) (*P*-heterogeneity = 0.33). In addition, a hospital-based case–control study reported an inverse association among women who underwent the procedure more than five years ago (RR = 0.37, 95%CI: 0.11-1.24), but no association among those who had a hysterectomy within five years (RR = 1.04, 95%CI: 0.37-2.90) [29]. Five studies examined age at hysterectomy on ovarian cancer risk, three dichotomized at age 40 and two at age 45. In a meta-regression, hysterectomy was more strongly inversely associated with ovarian cancer among women who were younger than 40 or 45 at surgery compared to 40 or 45 years of age or older, however the p for heterogeneity was not statistically significant (*P*-heterogeneity = 0.29). The summary RR for women less than 40 or 45 years of age was 0.70 (95%CI: 0.55, 0.89) compared to 0.83 (95%CI: 0.72, 0.96) for women over 40 or 45 years of age (Table 4).

## Discussion

Observational epidemiologic evidence strongly suggests that there is a decreased risk of ovarian cancer among women who have had a tubal ligation or hysterectomy. We observed an approximately 26-30% reduction in ovarian cancer risk among women who had a tubal ligation or hysterectomy compared to women who never had a tubal ligation or hysterectomy, respectively. These estimates did not vary substantially by study design or population. We did not observe any significant differences in the effect estimates by years since procedure. For both hysterectomy and tubal ligation, the inverse association between these procedures and ovarian cancer risk was suggestively stronger among women who underwent the procedure at earlier ages. There was evidence that tubal ligation may be associated with a stronger reduced risk for endometrioid tumors compared to serous tumors; however this finding was based on studies with small numbers of cases of each subtype and should be interpreted cautiously.

Several mechanisms have been proposed to explain the observed inverse association between tubal ligation and hysterectomy and ovarian cancer risk. One potential explanation is a “screening effect” wherein surgeons are able to visualize abnormal changes in the ovaries during tubal sterilizations or hysterectomies and remove pre-malignant lesions. If the inverse association was solely due to screening of the ovaries, these procedures would be associated with a lower risk for only a few years after the surgery; however this was not supported in our analysis as there was a strong inverse association even more than 10 years after surgery. Another potential mechanism is that tubal ligation and hysterectomy protect the ovary from carcinogens, such as talc, or inflammatory agents such as retrograde menstruation or endometriosis ascending the genital tract. Green et al. reported that ovarian cancer risk was highest among women who used talc and did not have a tubal ligation or hysterectomy and lowest among women who had surgical sterilization, but did not use talc [26]. However, in the Nurses’ Health Study (NHS), there was no variation in RR estimates of tubal ligation and ovarian cancer by talc use, and in a large case–control study, the inverse association of tubal ligation and hysterectomy was limited to non-talc users, contrary to the ascending carcinogen hypothesis [43,50].

Ovarian cancer risk may be altered by decreased blood supply to the ovary after surgery resulting in a decrease in estrogen production. However, while some studies have observed decreases in hormone levels after tubal ligation or hysterectomy, [51-53] others have not [54,55]. This mechanism may only apply to procedures that cause substantial damage to the surrounding tissue. In the NHS, women who had undergone tubal ligation during the time period when the unipolar electrocautery method was commonly used had a reduced risk of breast cancer [56]. However, tubal ligation was not associated with breast cancer risk during other periods when methods that caused less tissue destruction were common. To our knowledge, only one study examined ovarian cancer risk by type of tubal ligation and observed a lower risk irrespective of technique [26]. However this analysis was based on only 20 cases and 58 controls and thus had limited power. Lastly, several cancers, including ovarian cancers, over-express the surface glycoprotein MUC1. It has been hypothesized that women who have undergone events that trigger an immune response to MUC1 have a decreased risk of ovarian cancer [4]. A recent study reported higher anti-MUC1 antibodies were associated with a decreased risk of ovarian cancer among women less than 64 years of age [57]. In the same study, women who had undergone a tubal ligation had higher mean levels of anti-MUC1 antibodies compared to women who had not undergone a tubal ligation; however there were no differences in antibodies levels by hysterectomy status [57]. Further research is needed to determine the associations between surgical procedures, anti-MUC1 antibodies, and subsequent ovarian cancer risk.

Our analysis has several limitations. Not all studies reported whether cases were restricted to invasive ovarian cancer., however when we restricted to studies that reported effect estimates for invasive ovarian cancer the summary RRs were very similar. Few studies reported effect estimates by surgical characteristics or histological subtype of ovarian cancer. In addition, when reported, these stratum-specific estimates were often based on small numbers of exposed cases. To pool effect estimates for analysis of age at and years since tubal ligation, we created very broad categories (e.g., age at tubal ligation <35 years, ≥35 years; hysterectomy <10 years ago, ≥10 years ago), which may obscure important effects. Some of the studies in the meta-analysis included both prevalent as well as incident ovarian cancer cases and the case definition for one study was ovarian cancer mortality. If tubal ligation or hysterectomy were associated with survival after ovarian cancer diagnosis then the inclusion of prevalent cases may bias the effect estimates. However, a recent systematic review did not support an association between tubal ligation or hysterectomy and survival from ovarian cancer [58].

In summary, we observed a consistent inverse association of tubal ligation and hysterectomy on ovarian cancer risk that may be causal. We did not detect differences by study design, study population, or years since the procedure, although our statistical power in these analyses was somewhat limited. While gynecologic surgery may be a potential prevention strategy for women at high risk of ovarian cancer, additional research is needed to determine whether the effect of tubal ligation and hysterectomy on ovarian cancer risk differs by individual and surgical characteristics as well as considering the potential negative health effects of these procedures. Additional research also is needed to further understand the mechanisms behind these reduced risks.

## Competing interests

The authors declare that they have no competing interests.

## Authors’ contributions

MSR participated in the design of the study, conducted the literature search for all tubal ligation articles, extracted data, analyzed the data and authored the manuscript. MAM conducted the literature search for all hysterectomy articles and extracted data. SST participated in the design of the study, reviewed the data extracted, and helped draft the manuscript. All authors read and approved the final manuscript.

## Supplementary Material

Additional file 1 **Table S1, Table S2, Table S3, Table S4, Table S5.** Epidemiologic Studies of the Association Between Tubal Ligation and Risk of Ovarian Cancer by Years Since Procedure. Epidemiologic Studies of the Association Between Tubal Ligation and Risk of Ovarian Cancer by Age at Procedure. Epidemiologic Studies of the Association Between Tubal Ligation and Risk of Ovarian Cancer by Histological Subtype. Epidemiologic Studies of the Association Between Hysterectomy and Risk of Ovarian Cancer by Years Since Procedure. Epidemiologic Studies of the Association Between Hysterectomy and Risk of Ovarian Cancer by Age at Procedure [59]. Click here for file
