# Supplementary material for: Tubal ligation, hysterectomy and ovarian cancer: A meta-analysis
Source: J Ovarian Res. 2012 May 15;5:13. doi: 10.1186/1757-2215-5-13 (PMC3386864; doi:10.1186/1757-2215-5-13)
Supplement: Additional file 1 — Table S1, Table S2, Table S3, Table S4, Table S5. Epidemiologic Studies of the Association Between Tubal Ligation and Risk of Ovarian Cancer by Years Since Procedure. Epidemiologic Studies of the Association Between Tubal Ligation and Risk of Ovarian Cancer by Age at Procedure. Epidemiologic Studies of the Association Between Tubal Ligation and Risk of Ovarian Cancer by Histological Subtype. Epidemiologic Studies of the Association Between Hysterectomy and Risk of Ovarian Cancer by Years Since Procedure. Epidemiologic Studies of the Association Between Hysterectomy and Risk of Ovarian Cancer by Age at Procedure [59]. [file 1757-2215-5-13-S1.doc]

Supplemental Table 1: Epidemiologic Studies of the Association Between Tubal Ligation and Risk of Ovarian Cancer by Years Since Procedure

| **Author (Country)** | **Study Design** | **Case definition** | **Covariates** | **OR, RR, or SIR (95%CI), by years since procedure** | **Comments** |
| --- | --- | --- | --- | --- | --- |
| NECC 2012 (USA)  [Not published] | Case-control | Borderline or invasive epithelial ovarian cancer  <10 years since TL=37 cases  10+ years since TL = 239 cases | age, study center, BMI , study phase, smoking, family history of ovarian and breast cancers, talc use, OC use, parity, breast feeding, age at menarche, post-menopausal status, use of post-menopausal hormones, hysterectomy | <10: 0.67 (0.44, 1.02)  10+: 0.80 (0.66, 0.97) |  |
| Dorjgochoo T. et al. 2009 (China) [14] | Prospective cohort | Ovarian cancer  <33 years since TL=76 cases  ≥33 years since TL=18 cases | Age, education, age at menarche, parity, breastfeeding, BMI, physical activity, smoking, menopausal status, family history of cancer, other contraceptive methods. | <33: 1.10 (0.50, 2.40)  >33: 1.32 (0.55, 3.12) | Cohort N=66,661 |
| Kjaer et al. 2004 (Denmark) [19] | Population-based follow-up study | Invasive ovarian cancer  1-4 years since  TL=17 cases  5-9 years since  TL=37 cases  >10 years since  TL=19 cases  Number of cases by years since TL not provided for borderline cases | Age and calendar year | Invasive  <1: 0.43 (0.1, 1.6)  1-4: 0.71 (0.4, 1.1)  5-9: 1.08 (0.8, 1.5)  >10: 0.65 (0.4, 1.0)  Borderline  <1: not calculated  1-4: 0.94 (0.4, 1.9)  5-9: 0.84 (0.4, 1.7)  >10: 0.86 (0.3, 1.9) | Observed number of cancer cases in cohort of women who underwent tubal ligation was compared to the expected number of cases based on the age and calendar year specific rates from the Danish Cancer Registry. |
| Green, et al. 1997 (Australia) [26] | Case-control | Incident, primary epithelial ovarian cancer  0-4 years since TL= 9 cases  5-9 years since TL = 14 cases  10-14 years since TL = 29 cases  15-19 years since TL = 36 cases  20-24 years since TL = 8 cases  25+ years since TL = 8 cases | Age, education, BMI, parity, OC duration, smoking, family history of ovarian cancer | 0-4: 0.42 (0.19,0.96)  5-9: 0.56 (0.27,1.1)  10-14: 0.72 (0.43,1.2)  15-19: 0.98 (0.60,1.6)  20-24: 0.26(0.11,0.62)  25+: 0.43 (0.18,1.0) | 90% participation rate in cases, 73% in controls. |
| Miracle-McMahill, et al. 1997 (USA) [28] | Prospective Cohort Study | Ovarian cancer mortality  <20 years since TL=8 ovarian cancer deaths  ≥20 years since TL=15 ovarian cancer deaths | Age, race, BMI, education, family history of ovarian cancer, family history of breast cancer, parity, marital status,  age at menarche, OC use, ERT, age at menopause, miscarriages  smoking status | <20: 0.49 (0.24,0.99)  > 20: 0.80 (0.48,1.34) |  |
| Kreiger et al, 1997 (Canada) [25] | Historical cohort study | Ovarian cancer  6mo-2yrs since TL = 19 cases  2-<5 years since TL = 32 cases  5-<10 years since TL= 44 cases  10+ years since TL = 13 cases | Age, calendar year, length of follow-up | 6mo-2yrs:  0.52 p=0.002  2-<5:  0.69 p=0.036  5-<10:  0.63 p=0.001  10+:  0.36 p<0.001 |  |
| Rosenblatt, et al. 1996 (International) [29] | Case-control | Borderline or malignant epithelial ovarian cancer  1-5 years since TL = 9 cases  6-10 years since TL = 8 cases  11-15 years since TL = 5 cases  16-20 years since TL = 2 cases  >20 years since TL = 10 cases | Age, hospital, year of interview, parity  OC use | 1-5: 0.96 (0.46,1.98)  6-10: 0.58 (0.28,1.24)  11-15: 0.60(0.23,1.52)  16-20: 0.27(0.06,1.15)  >20: 1.44 (0.66,3.15) | No differences observed for borderline and malignant tumors. ORs for clear cell and endometrioid based on one case each. |
| Irwin et al. 1991 (USA) [48] | Case-control | Epithelial ovarian cancer  <4 years since TL = 16 cases  5-9 years since TL = 14 cases  10-14 years since TL = 6 cases  15-19 years since TL = 4 cases  20+ years since TL = 7 cases | Age and parity | <4:  0.69 (0.41-1.17)  5-9:  0.55 (0.32-0.96)  10-14:  0.53 (0.23-1.22)  15-19:  1.03 (0.36-2.92)  20+:  1.44 (0.63-3.27) |  |

Abbreviations: OR, odds ratio; RR, relative risk; SIR, standardized incidence ratio; OC, oral contraceptive; BMI, body mass index; ERT, estrogen replacement therapy; TL, tubal ligation

Supplemental Table 2: Epidemiologic Studies of the Association Between Tubal Ligation and Risk of Ovarian Cancer by Age at Procedure

| **Author (Country)** | **Study Design** | **Case definition** | **Covariates** | **OR, RR, or SIR (95%CI)** | **Comments** |
| --- | --- | --- | --- | --- | --- |
| NECC 2012 (USA)  [Not published] | Case-control | Borderline or invasive epithelial ovarian cancer  <35 years at TL =144 cases  35+ years at TL = 135 cases | age, study center, BMI , study phase, smoking, family history of ovarian and breast cancers, talc use, OC use, parity, breast feeding, age at menarche, post-menopausal status, use of post-menopausal hormones, hysterectomy | <35: 0.74 (0.59, 0.94)  35+: 0.84 (0.66, 1.07) |  |
| Antoniou et al. 2009 (Europe and Canada) [13] | Retrospective Cohort | Ovarian cancer (only BRCA 1/2 carriers)  ≤35 years at TL = 9 cases  >35 years at TL = 14 cases | Age, duration of OC use, parity | Never: 3.40 (1.45, 7.99)  ≤35: 1.00  >35: 2.00 (0.68, 5.86) | Includes prevalent as well as incident cases.  Mean difference between age at diagnosis and interview: 6.7 years |
| Dorjgochoo T. et al. 2009 (China) [14] | Prospective cohort | Ovarian cancer  <30 years at TL=11 cases  ≥30 years at TL=7 cases | Age, education, age at menarche, parity, breastfeeding, BMI, physical activity, smoking, menopausal status, family history of cancer, other contraceptive methods. | <30: 1.40 (0.65, 3.04)  ≥30: 0.98 (0.42, 2.32) | Cohort N=66,661 |
| Kjaer et al. 2004 (Denmark) [19] | Population-based follow-up study | Invasive ovarian cancer and borderline ovarian tumor  <35 years at TL= number of cases not provided  ≥35 years at TL= number of cases not provided  Cohort N=65,236 | Age and calendar year | <35: 0.78 (0.5, 1.2)  >35: 0.83 (0.6, 1.1) | Observed number of cancer cases in cohort of women who underwent tubal ligation was compared to the expected number of cases based on the age and calendar year specific rates from the Danish Cancer Registry. |
| Cornelison et al 1997 (USA) [27] | Case-control | Ovarian cancer  < 24 years at TL = 1 case  25-34 years at TL = 11 cases  35-44 years at TL = 12 cases  45-58 years at TL = 2 cases | Age , SES, marital status, parity, age at first pregnancy, age at menarche, age at menopause, irregular menses, breast-feeding duration, BMI, OC use | < 24: 0.20 (0.01,1.55)  25-34: 0.44 (0.21,0.92)  35-44: 0.63 (0.30,1.30)  45-58: 0.89 (0.12,5.74) | Patient controls with no malignancy or ovarian disease. |
| Miracle-McMahill, et al. 1997 (USA) [28] | Prospective Cohort Study | Ovarian cancer mortality  <35 years at TL=11 ovarian cancer deaths  ≥35 years at TL=12 ovarian cancer deaths | Age, race, BMI, education, family history of ovarian ca, family history of breast cancer, parity, marital status,  age at menarche, OC use, ERT, age at menopause, miscarriages  smoking status | <35: 0.71 (0.34,1.30)  >35: 0.62 (0.34,1.10) |  |
| Kreiger et al, 1997 (Canada) [25] | Historical cohort study | Ovarian cancer  15-24 years at TL = 8 cases  25-44 years at TL = 90 cases  45-64 years at TL = 10 cases | Age, calendar year, length of follow-up | 15-24:  0.63 p=0.220  25-44:  0.54 p<0.001  45-64:  1.18 p=0.678 |  |
| Rosenblatt, et al. 1996 (International) [29] | Case-control | Borderline or malignant epithelial ovarian cancer  < 27 years at  TL = 11 cases  28-30 years at TL = 7 cases  31-35 years at TL = 8 cases  >35 years at  TL = 8 cases | Age, hospital, year of interview, parity  Oral contraceptive use | < 27: 0.92 (0.46,1.84)  28-30: 0.76 (0.33,1.69)  31-35: 0.77 (0.36,1.64)  >35: 0.59 (0.28,1.24) |  |
| Irwin et al. 1991 (USA) [48] | Case-control | Epithelial ovarian cancer  Age <35:  number of cases not provided  Age 35+:  number of cases not provided | Age and parity | Age <35 and ≤9 year prior: 0.45 (0.24-0.84)  Age <35 and >9 year prior: 0.79 (0.42-1.44)  Age 35+ and ≤9 year prior: 0.79 (0.48-1.28)  Age 35+ and >9 year prior: 1.05 (0.41-2.68) |  |

Abbreviations: OR, odds ratio; RR, relative risk; SIR, standardized incidence ratio; TL, tubal ligation; OC, oral contraceptives; ERT, estrogen replacement therapy, BMI, body mass index

Supplemental Table 3: Epidemiologic Studies of the Association Between Tubal Ligation and Risk of Ovarian Cancer by Histological Subtype

| **Author (Country)** | **Study Design** | **Case definition** | **Covariates** | **OR, RR, or SIR (95%CI)** | **Comments** |
| --- | --- | --- | --- | --- | --- |
| NECC 2012 (USA)  [Not published] | Case-control | Borderline or invasive epithelial ovarian cancer  N=1114 serous cases  N= 239 mucinous cases  N=324 endometrioid cases  N=259 clear cell cases  N=140 other cases | age, study center, BMI , study phase, smoking, family history of ovarian and breast cancers, talc use, OC use, parity, breast feeding, age at menarche, post-menopausal status, use of post-menopausal hormones, hysterectomy | Serous:  0.91 (0.74, 1.12)  Mucinous:  0.80 (0.52, 1.25)  Endometrioid:  0.40 (0.25, 0.63)  Clear cell:  0.71 (0.47, 1.09)  Other:  0.82 (0.49, 1.38) |  |
| Nagle et al. 2008 (Australia) [58] | Case-control | Invasive epithelial ovarian cancer  N=142 endometrioid cases  N=90 clear cell cases | Age, education, parity, and hormone/ contraceptive use | Endometrioid:  0.4 (0.3, 0.7)  Clear cell:  0.7 (0.4, 1.2) | 47% participation rate in controls |
| Jordan et al.  2008  (Australia) [10] | Case-control | Invasive serous ovarian cancer  N=627 invasive serous | Parity, hormonal contraceptive use, history of breast or ovarian cancer, age, education | Serous (invasive):  0.87 (0.69-1.09) |  |
| Jordan et al. 2007  (Australia) [16] | Case-control | N=230 benign serous tumors  N=133 benign mucinous tumors | Age, state of residence, education, parity, hormonal contraceptive use, hysterectomy, smoking status | Mucinous:  1.00 (0.61-1.64)  Serous:  1.08 (0.75-1.57) | 65% participation rate in cases, 47% in controls. |
| Kjaer et al. 2004 (Denmark) [19] | Population-based follow-up study | Invasive and borderline ovarian cancer  N=26 serous cases  N=19 mucinous cases  N=14 clear cell/ endometrioid cases  N=16 other cases | Age and calendar year | Serous:  0.72 (0.5, 1.1)  Mucinous:  1.49 (0.9, 2.3)  Clear cell/ endometrioid:  0.86 (0.5, 1.4)  Other:  0.60 (0.3, 1.0) | Observed number of cancer cases in cohort of women who underwent tubal ligation was compared to the expected number of cases based on the age and calendar year specific rates from the Danish Cancer Registry. |
| Tung et al. 2003  (USA) [49] | Case-control | Epithelial ovarian cancer  Invasive  N=48 mucinous cases  N=220  serous cases  N=72  endometrioid cases  N=48  clear cell cases  N=43 other cases  Borderline  N=61 mucinous cases  N=66  serous cases | Age, ethnicity, study site, education, pregnancy status, OC use | Invasive  Mucinous:  0.9 (0.4, 2.1)  Serous:  0.8 (0.5, 1.2)  Endometrioid: 0.2 (0.1, 0.6)  Clear cell:  0.5 (0.2, 1.4)  Other:  1.2 (0.5, 2.9)  Borderline  Mucinous:  1.1 (0.5, 2.3)  Serous:  0.6 (0.3, 1.4) | Same study population as a case-control study included in Modugno et al. 2004 |
| Modugno et al. 2001 (USA) [59] | Case-control | Epithelial ovarian cancer  N=357 serous cases  N=112 mucinous cases  N=139 endometrioid cases  N=159 other cases | Age, number of live births, years of OC use, years of non-contraceptive estrogen use, months breastfed, hysterectomy, family history, ethnicity | Serous:  0.52 (0.38, 0.72)  Mucinous:  0.47 (0.27, 0.82)  Endometrioid:  0.48 (0.28, 0.78)  Other:  0.76 (0.49, 1.17) | Same study population as a case-control study included in Modugno et al. 2004  Includes borderline and invasive cases. |
| Wittenberg et al. 1999 (USA) [24] | Case-control | Epithelial ovarian cancer  N=43 mucinous cases  N=279 non-mucinous cases | Age at diagnosis, parity, duration of OC use | Mucinous:  0.4 (0.1, 1.9)  Non-mucinous:  0.6 (0.3, 1.1) | 64% participation rate in cases, 72% in controls. Included both borderline and invasive |
| Green, et al. 1997 (Australia) [26] | Case-control | Incident, primary epithelial ovarian cancer  Number of serous cases not reported | Age, education, BMI, parity, OC duration, smoking, family history of ovarian cancer | Serous:  0.54 (0.42, 0.70) | 90% participation rate in cases, 73% in controls. |
| Risch et al.  1996 (Canada) [22] | Case-control | Epithelial ovarian cancer  Invasive  N=212 serous cases  N=40 mucinous cases  N=73 endometrioid cases  N=42 other cases  Borderline:  N=42 serous cases  N=40 mucinous cases | Age, parity, years of OC use, average lactation/pregnancy, total years of ERT, hysterectomy, family history of breast cancer | Invasive  All:  0.58 (0.39-0.85)  Serous:  0.60 (0.38-0.97)  Mucinous:  0.48 (0.20-1.16)  Endometrioid:  0.49 (0.23-1.07)  Other:  0.85 (0.36-1.99)  Borderline:  All:  1.12 (0.64-1.95)  Serous:  1.58 (0.74-3.39)  Mucinous:  0.86 (0.39-1.90)  All:  0.67 (0.47-0.94)  Serous:  0.75 (0.49-1.14)  Mucinous:  0.64 (0.35-1.17) |  |
| Rosenblatt, et al. 1996 (International) [29] | Case-control | Borderline or malignant epithelial ovarian cancer  N=17 serous cases  N=15 mucinous cases  N=1 clear cell case  N=1 endometrioid case | Age, hospital, year of interview, parity,  OC use | Serous:  0.98 (0.53, 1.80)  Mucinous:  0.88 (0.46, 1.69)  Clear cell:  0.33 (0.01, 2.68)  Endometrioid:  0.21 (0.05, 1.49) |  |

Abbreviations: OR, odds ratio; RR, relative risk; SIR, standardized incidence ratio; OC, oral contraceptive; BMI, body mass index

Supplemental Table 4: Epidemiologic Studies of the Association Between Hysterectomy and Risk of Ovarian Cancer by Years Since Procedure

| Author (Country) | Study Design | Case definition | Covariates | OR, RR, or SIR (95%CI) | Comments |
| --- | --- | --- | --- | --- | --- |
| NECC (USA) [Not published] | Case-control study | Epithelial ovarian cancer  <10 years since hyst= 38 cases  10+ years since hyst= 147 cases | age, study center , BMI , study phase, smoking, family history of ovarian and breast cancers, talc use, OC use , parity, breast feeding, age at menarche, post-menopausal status, use of post-menopausal hormones tubal ligation | Hyterectomy with or without unilateral oophorectomy:  <10 years since hyst: 0.83 (0.53-1.30)  10+ years since hyst:  0.99 (0.76-1.30) |  |
| Kreiger et al. 1997  (Canada) [25] | Historical cohort study | Invasive and borderline ovarian cancer  6mo-<2yrs since hyst = 24 cases  2-<5 since hyst = 53 cases  5-<10 since hyst = 68 cases  10+ since hyst = 24 cases | Age, calendar year, length of follow-up | Hysterectomy only:  6mo-<2yrs: 0.50 p<0.01  2-<5: 0.78 p=0.072  5-<10: 0.79 p=0.046  10+: 0.74 p=0.157 | Calculated observed over expected events. |
| Green, et al. 1997 (Australia) [26] | Case-control | Incident, primary epithelial ovarian cancer  0-4 years since hyst= 15 cases  5-9 years since hyst = 18 cases  10-14 years since hyst = 22 cases  15-19 years since hyst = 19 cases  20-24 years since hyst = 17 cases  25+ years since hyst = 25 cases | Age, education, BMI, parity, OC duration, smoking, family history of ovarian cancer | Hysterectomy with or without unilateral oophorectomy:  0-4: 1.5 (0.73-3.3)  5-9: 0.89 (0.45-1.7)  10-14: 0.67 (0.37-1.2)  15-19: 0.52 (0.28-0.94)  20-24: 0.54 (0.28-1.1)  25+: 0.49 (0.28-0.89) | 90% participation rate in cases, 73% in controls. |
| Hankinson et al. 1993 (USA) [43] | Cohort study (NHS) | Borderline and malignant epithelial ovarian cancer  1-4 years since hyst = 5 cases  5-9 years since hyst = 3 cases  10-14 years since hyst =11 cases  15+ years since hyst = 9 cases | Age, parity, duration of OC use, age at menarche, tubal ligation, smoking status, BMI | Hysterectomy only:  1-4: 1.03 (0.42-2.51)  5-9: 0.29 (0.10-0.88)  10-14: 0.92 (0.50-1.69)  15+: 0.62 (0.31-1.22) |  |
| Parazzini et al. 1993 (Italy) [45] | Case-control study | Epithelial ovarian cancer  <5 years since hyst = 12 cases  5-9 years since hyst = 9 cases  10-14 years since hyst = 10 cases  15+ years since hyst = 13 cases | Age, education, parity, lifelong menstrual pattern, oral contraceptive use, | Hysterectomy only:  <5: 0.9 (0.4-1.7)  5-9: 0.7 (0.3-1.6)  10-14:0.7 (0.3-1.4)  15+:0.5 (0.3-0.8) |  |
| Risch et al. 1994  (Canada) [46] | Case-control | Epithelial ovarian cancer  0-5 years since hyst = 7 cases  6-10 years since hyst = 12 cases  11-15 years since hyst = 13 cases  16-20 years since hyst = 7 cases  21-25 years since hyst = 8 cases  >25 years since hyst = 15 cases | Age, duration of OC use, number of full-term pregnancies | Hysterectomy with or without unilateral oophorectomy:  0-5: 0.56 (0.22-1.45)  6-10: 0.53 (0.26-1.08)  11-15: 0.70 (0.33-1.46)  16-20: 0.42 (0.17-1.02)  21-25: 0.35 (0.16-0.80)  >25: 0.53 (0.28-1.02) |  |
| Rosenblatt et al. 1996  (7 countries) [29] | Case-control (Multi-site/country) | Borderline or invasive epithelial ovarian cancer  1-5 years since hyst = 5 cases  >5 years since hyst = 3 cases | Age, date of diagnosis, center, parity, OC use | Hysterectomy with or without unilateral oophorectomy:  1-5: 1.04 (0.37-2.90)  >5: 0.37 (0.11-1.24) | By time since and age at |
| Whittemore et al 1992  (USA) [31] | Pooled case-control | Invasive epithelial ovarian cancer  Hospital-based studies:  2-9 years since hyst = 26 cases  10-19 years since hyst = 27 cases  20+ years since hyst = 32 cases  Population-based studies:  2-9 years since hyst = 52 cases  10-19 years since hyst = 62 cases  20+ years since hyst = 41 cases | Age, study, parity, OC use | Hysterectomy with or without unilateral oophorectomy:  Hospital-based studies:  2-9: 0.64 (0.40-1.00)  10-19: 0.52 (0.33-0.81)  20+: 0.86 (0.55-1.3)  Population-based studies:  2-9: 0.99 (0.71-1.4)  10-19: 0.86 (0.63-1.2)  20+: 0.79 (0.53-1.2) | Restricted to white women. 6 hospital based studies and 6 population-based studies. All hysterectomies performed at least 2 years prior to reference date. |

Abbreviations: OR, odds ratio; RR, relative risk; SIR, standardized incidence ratio; OC, oral contraceptive; BMI, body mass index; TL, tubal ligation

Supplemental Table 5: Epidemiologic Studies of the Association Between Hysterectomy and Risk of Ovarian Cancer by Age at Procedure

| Author (Country) | Study Design | Case definition | Covariates | OR, RR, or SIR (95%CI) | Comments |
| --- | --- | --- | --- | --- | --- |
| NECC (USA) [Not published] | Case-control | Epithelial ovarian cancer  <40 years at hyst = 98 cases  40+ years at hyst = 87 cases | age, study center, BMI, study phase, smoking, family history of ovarian and breast cancers, talc use, OC use , parity, breast feeding, age at menarche, post-menopausal status, use of post-menopausal hormones, tubal ligation | Hysterectomy with or without oophorectomy:  <40: 1.12 (0.80-1.55)  45+: 0.82 (0.60-1.12) |  |
| Kreiger et al. 1997  (Canada) [25] | Historical cohort study | Ovarian cancer  15-24 years at hyst = 0 cases  25-44 years at hyst = 54 cases  45-64 years at hyst = 82 cases  65+ years at hyst = 33 cases | Age, calendar year, length of follow-up | Hysterectomy only:  15-24: 0.00 p=0.993  25-44: 0.55 p<0.001  45-64: 0.90 p=0.370  65+: 0.73 p=0.066 | ***Also has TL  Calculated observed over expected events. |
| Hankinson et al. 1993 (USA) [43] | Cohort study (NHS) | Borderline and invasive epithelial ovarian cancer  <45 years at hyst = 6 cases  45+ years at hyst = 22 cases | Age, parity, duration of OC use, age at menarche, tubal ligation, smoking status, BMI | Hysterectomy only:  <45: 0.48 (0.21-1.08)  45+: 0.76 (0.48-1.19) |  |
| Rosenblatt et al. 1996  (7 countries) [29] | Case-control (Multi-site/country) | Borderline or malignant epithelial ovarian cancer  ≤40 years at hyst = 4 cases  >40 years at hyst = 4 cases | Age, date of diagnosis, center, parity, OC use | Hysterectomy with or without unilateral oophorectomy:  ≤40: 0.50 (0.17-1.50)  >40: 0.78 (0.25-2.40) |  |
| Whittemore et al 1992  (USA) [31] | Pooled case-control | Invasive epithelial ovarian cancer  Hospital-based studies:  <40 years at hyst = 38 cases  40+ years at hyst = 47 cases  Population-based studies:  <40 years at hyst = 65 cases  40+ years at hyst = 90 cases | Age, study, parity, OC use | Hysterectomy with or without unilateral oophorectomy:  Hospital-based studies:  <40: 0.58 (0.40-0.86)  40+: 0.73 (0.51-1.0)  Population-based studies:  <40: 0.76 (0.57-1.00)  40+: 1.0 (0.77-1.3) | Restricted to white women. 6 hospital based studies and 6 population-based studies. All hysterectomies performed at least 2 years prior to reference date. |

Abbreviations: OR, odds ratio; RR, relative risk; SIR, standardized incidence ratio; TL, tubal ligation; OC, oral contraceptives; BMI, body mass index
